# A Study of a Novel Disposable Endoscopic Purse‐String Suture Auxiliary Instrument for the Treatment of Full‐Thickness Gastric Wall Defects

**DOI:** 10.1111/1751-2980.13338

**Published:** 2025-04-15

**Authors:** Ying Zhou, Bai Sheng Chen, Qi Jiang, Na Shan Li, Pei Hong Zhang, Dan Feng Zhang, Yuan Ling Ruan, Ping Li, Xia Wu, Ping Hong Zhou, Wei Feng Chen

**Affiliations:** ^1^ Department of Endoscopic Center, Zhongshan Hospital Fudan University (Xiamen Branch), Xiamen Clinical Research Center for Cancer Therapy Xiamen Fujian Province China; ^2^ Department of Endoscopic Center Zhongshan Hospital, Fudan University Shanghai China

**Keywords:** endoscopic purse‐string suture auxiliary instrument, operative surgical procedures, porcine stomach, success rate, suture time

## Abstract

**Objectives:**

In this study, we aimed to evaluate the effectiveness of a novel endoscopic purse‐string suture auxiliary instrument compared with traditional methods for closure of a full‐thickness defect of the stomach in an ex vivo model.

**Methods:**

Twelve perforation sites (10–20 mm in diameter) were created in the ex vivo porcine stomach models. Two physicians (A and B had performed endoscopic surgery for 6 and 3 years) performed suturing using both the experimental and traditional (control) instruments. Operation time, success rate, and number of attempts for successful suture required were recorded.

**Results:**

For physician A, the median suturing time was 56.50 s (interquartile range [IQR] 40.50 s, 134.50 s) and 215.50 s (IQR 63.75 s, 254.75 s) in the experimental and control groups. For physician B, they were 53.00 s (IQR 38.50 s, 87.75 s) and 174.00 s (IQR 104.50 s, 279.25 s), respectively. The differences between experimental and control groups were statistically significant for both physicians A (*p* = 0.010) and B (*p* = 0.004). The median number of attempts required for successful suturing in the experimental and control groups was 1 (IQR 1, 2) and 2 (IQR 1, 3) for physician A, and 1 (IQR 1, 1) and 3 (IQR 2, 3) for physician B, which were statistically significant for both physicians (*p* = 0.026 and 0.006). The overall success rate was significantly higher in the experimental group (100% vs. 75.0%, *p* = 0.022).

**Conclusion:**

This novel purse‐string suture auxiliary instrument may assist in single‐channel endoscopic suturing operations, improve the suture success rate, reduce the number of operations required, and shorten the operation time.

## Graphical abstract

We conducted a full perforation in the ex vivo porcine stomach model, which was closed with a novel endoscopic purse‐string suture auxiliary instrument and a conventional suture instrument, respectively. The effectiveness and feasibility of the novel auxiliary instrument were evaluated. It was found that this device could accelerate the single‐channel endoscopic suturing procedure, improve the success rate of suturing, reduce the number of procedures, and shorten the procedure time.

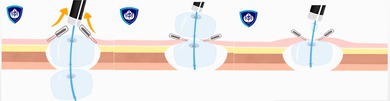

## 1 Introduction

Thanks to the development of endoscopic resection techniques in recent years, gastrointestinal diseases have been increasingly treated by using endoscopy in a minimally invasive manner. Full‐thickness defects of the digestive tract wall are common after endoscopic operations. Endoscopic full‐thickness resection (EFTR), natural orifice transluminal endoscopic surgery (NOTES), and other endoscopic procedures also actively incise the full thickness of the local digestive tract wall. Endoscopic purse‐string suture, metal clip closure suture, and particular suturing devices, such as over‐the‐scope clips (OTSCs), are generally used for repairing full‐thickness defects [1, 2, 3, 4, 5, 6]. Multiple titanium clips have been used to fix the nylon rope on the mucosa surrounding a full‐thickness defect, and the nylon rope is tightened to gather the surrounding mucosa. This suture method is less expensive, simple, and convenient to operate, and is widely applied in clinical practice.

In a clinical setting, it is necessary to adjust the direction of metal titanium clips before tightening the nylon rope to avoid the clips flipping towards the abdominal cavity or colliding with each other, which can negatively affect wound closure, and prevent metal titanium clips from falling into the abdominal cavity in the long term. Generally, biopsy forceps and foreign body forceps are used to adjust the direction of the titanium clips; however, these methods require high clinical skills and are time‐consuming. The increasing defect areas and number of the clips required also increase the uncontrollability of and difficulty in performing these procedures. The disposable endoscopic purse‐string suture auxiliary instrument is assisted by a balloon, which prevents the metal clips from flipping towards the abdominal cavity and effectively lowers the requirement of manual adjustment of the direction of the metal clips, thereby improving operating efficiency.

In this study, we used the ex vivo porcine stomach to establish the full‐thickness defect model, which was closed by purse‐string suturing under single‐channel endoscopy, and compared the effectiveness and feasibility of this novel endoscopic purse‐string suture auxiliary instrument with those of the traditional suture instrument.

## 2 Materials and Methods

### 2.1 Operators

Two physicians (physician A: B.S.C., and physician B: Y.Z.) performed all endoscopic procedures. The two physicians had performed endoscopic surgery for GI diseases for 6 and 3 years, respectively. In addition, a nurse assisted with the suturing operation.

### 2.2 Ex Vivo Model

Two fresh porcine stomachs were purchased from the market and an ex vivo porcine stomach endoscopic submucosal dissection (ESD) operation model (Erbe, Shanghai, China) was used. Briefly, both fresh porcine stomachs were used to establish a full‐thickness gastric defect model, in which 12 full‐thickness defects of approximately 10–20 mm in diameter were made at the antrum, body, and fundus of the stomach with a high‐frequency hook knife (KD‐620LR; Olympus, Tokyo, Japan) under a direct endoscope.

### 2.3 Study Design

**FIGURE 1:**
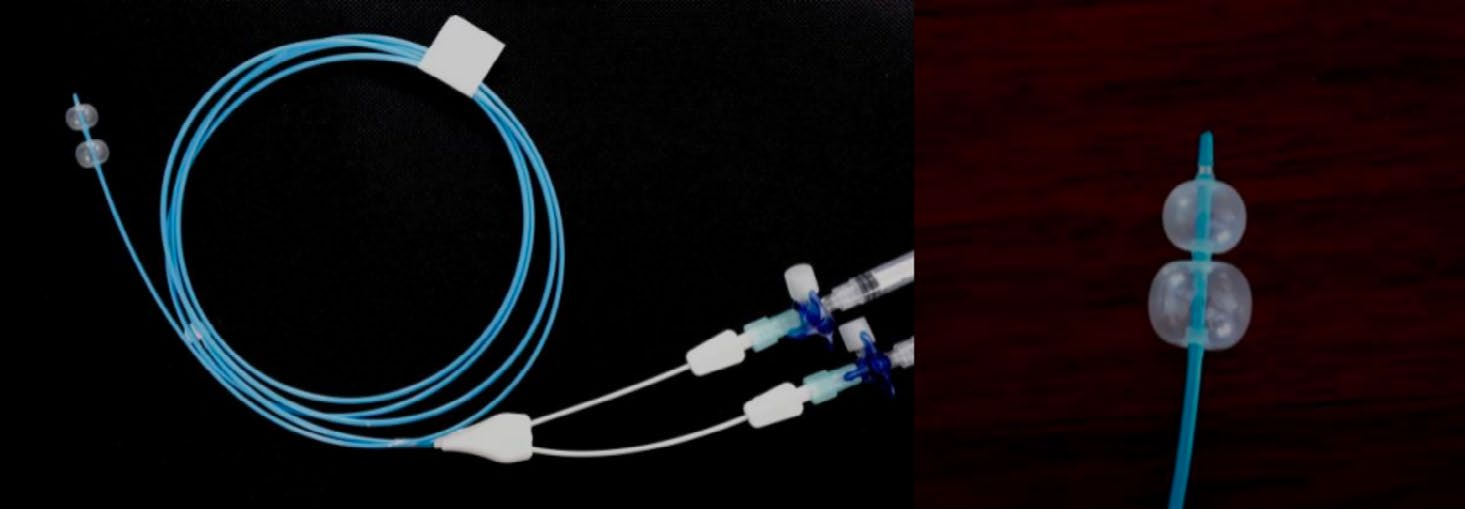
The novel purse‐string suture auxiliary instrument used for suturing operation.

**FIGURE 2:**
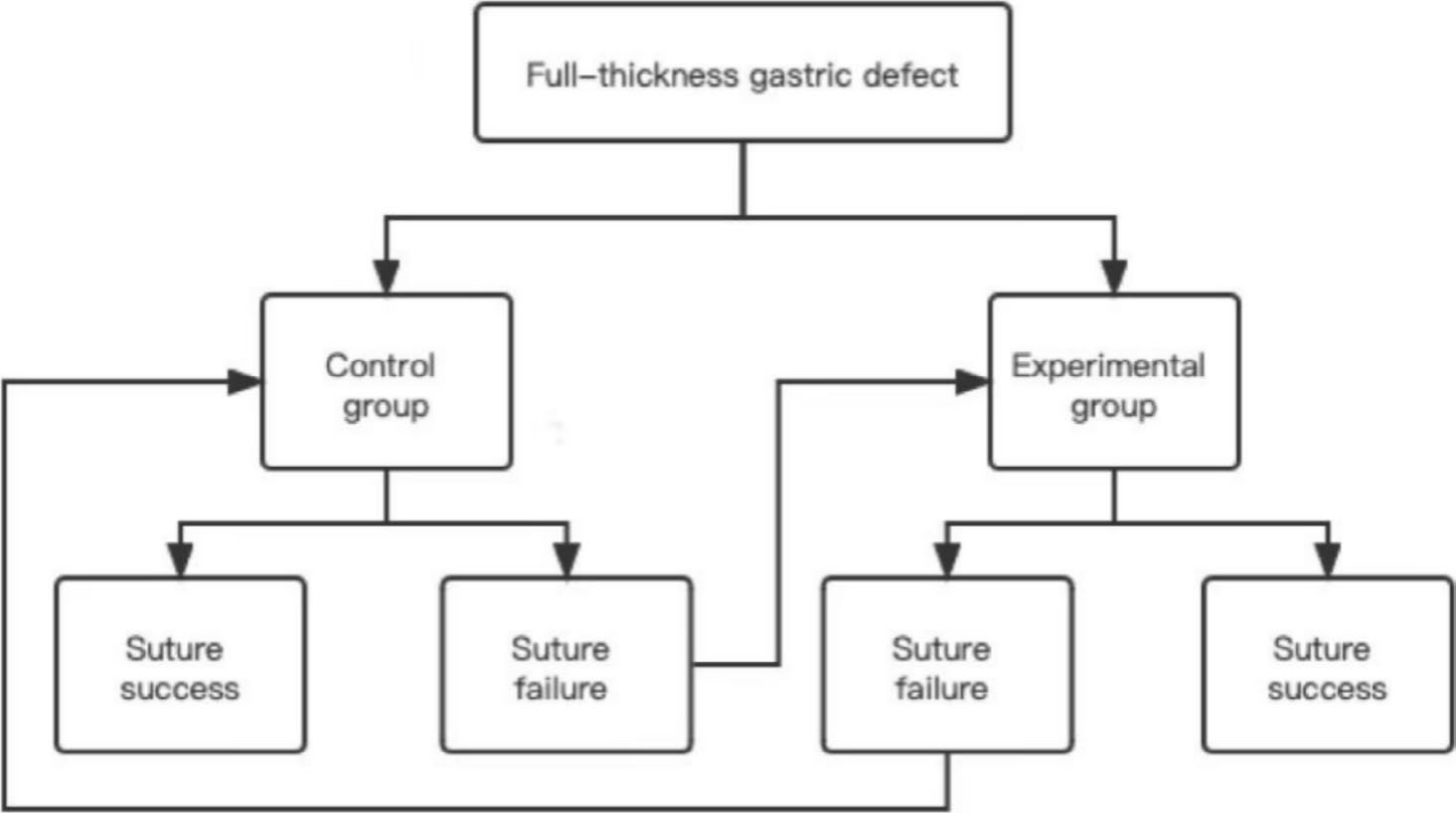
Flowchart of the operations.

The suturing operations performed on porcine stomach models were classified into the experimental and control groups. For closure of the artificial perforations, a novel purse‐string suture auxiliary instrument, which comprises an anterior balloon, a posterior balloon, a hollow catheter connecting the two balloons, and a syringe for air injection into the catheter, and the three‐way valve to control balloon inflation and deflation (Figure 1), was used in the experimental group, while in the control group the traditional purse‐string suture technique was used. First, management of the perforation sites using minimally invasive rotatable metal clips (ROCC‐D‐26‐195; MICRO‐TECH, Nanjing, Jiangsu Province, China) and nylon rope (MAJ‐340; Olympus) fixation was performed through endoscopic manipulation using the gastroscope (GIF‐H260) with a transparent distal attachment cap (D‐206‐05) (both from Olympus). Subsequently, each physician conducted one suturing operation for both the experimental and control groups for each perforation site. The four types of operations included: (a) physician A using the novel purse‐string suture auxiliary instrument for the experimental group; (b) physician A using the traditional purse‐string suture technique for the control group; (c) physician B using the novel purse‐string suture auxiliary instrument for the experimental group; and (d) physician B using the traditional purse‐string suture technique for the control group. The first three operations only involved pre‐tightening of the nylon rope. After recording the experimental indicators, the pre‐tightened nylon rope was released, and manual assistance was provided to restore the perforation site, metal clips, and nylon rope to their original status, while the tightening and release operation of the nylon rope was performed during the final suturing procedure only. The high‐frequency electrosurgical system (VIO200D; Erbe, Tübingen, Germany) and the ligating device (HX‐20Q‐1; Olympus) were used. The flowchart of the operations is shown in Figure 2.

The metal clips were ensured to be not everted or disorganized, and the perforation was closed after tightening of the nylon rope, which was regarded as a successful suture. Suture failure was considered when the perforation could not be completely closed with three consecutive sutures, after which the other suture method was attempted (Figure 2). According to the results of our preliminary experiment, the suturing time was (88 ± 41) s in the experimental group and (189 ± 76) s in the control group. The data of the preliminary experiment was analyzed using the Power Analysis & Sample Size (PASS) software version 15.0 (NCSS, Kaysville, UT, USA). Therefore, 12 perforation sites were adopted in the two porcine stomachs, with each perforation ranging from 10 to 20 mm in diameter. The perforation sites were located in the upper part (*n* = 1), middle part (*n* = 2), and the lower part of the gastric body (*n* = 3), the lesser curvature (*n* = 1), gastric fundus (*n* = 2), and gastric antrum (*n* = 3), respectively.

### 2.4 Procedures

In the control group, a conventional purse‐string suture technique was employed for the closure of the artificial perforation. The nylon rope was secured to the attachment cap of the gastroscope at one end and with a release device positioned parallel to the endoscope shaft, which is located external to the working channel, to advance into the gastric cavity. The assistant nurse operated the external release device to adjust the tension and position (relative to the perforation site) of the nylon suture. Hemoclips were deployed through the endoscopic working channel to secure the nylon suture circumferentially around the perforation site. Initial tension was applied to the suture under direct endoscopic visualization. The orientation of the clips was adjusted using biopsy forceps (Alton, Shanghai, China) throughout the procedure. After the confirmation of proper clip placement and verification of the absence of inversion into the abdominal cavity or interference between clips, the nylon suturing was tightened and released.

**FIGURE 3:**
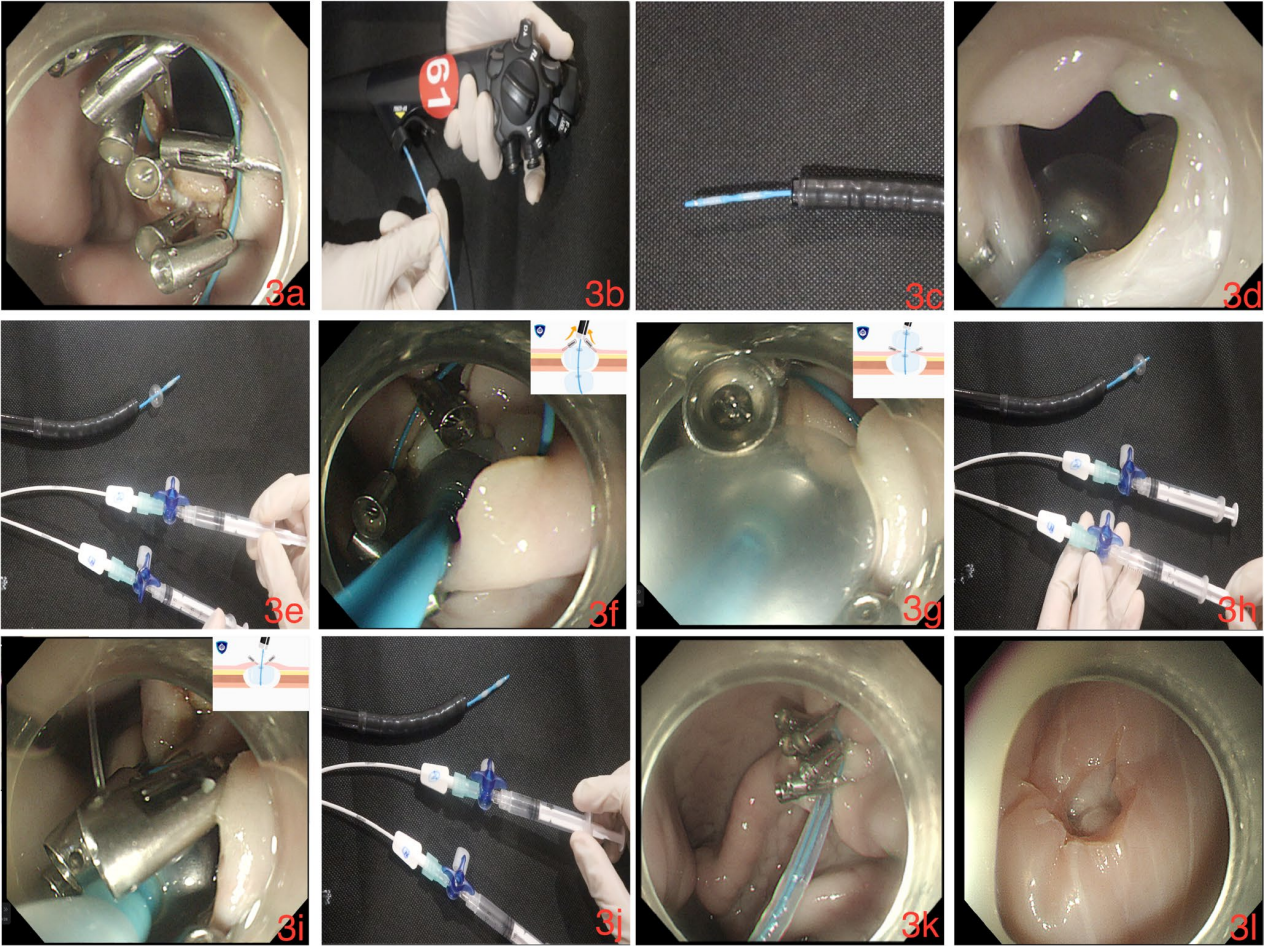
(a) The metal clips are used to fix the nylon rope evenly on the edge of the perforation site. (b, c) The novel disposable single‐channel endoscopic purse‐string suture auxiliary instrument is passed through the endoscopic forceps channel. (d) The balloon is pushed from inside the gastric cavity to the outside the stomach through the middle of the perforation. (e) Air (1.5 mL) is injected into both balloons through the three‐way value till they reach the diameter of about 1 cm. (f) The balloon drives the clips towards the gastric cavity, causing the clips to move gradually towards the gastric cavity. (g) The two arms of the metal clips, the gastric wall tissues grabbed with the arms, and the nylon rope are caught between the anterior and posterior balloons. (h) Air is withdrawn from the posterior balloon. (i) The anterior balloon remains inflated. (j) If the metal clips are not everted or disorganized, air is withdrawn from the anterior balloon. (k, l) The nylon loop is tightened to close the perforation.

While in the experimental group, a novel disposable single‐channel endoscopic purse‐string suture auxiliary instrument was used. The metal clips were used to evenly fix the nylon rope on the edge of the perforation site (Figure 3a), after which the novel instrument was passed through the endoscopic forceps channel (Figure 3b,c). The balloon was then pushed from the gastric cavity to outside the stomach through the middle of the perforation site (Figure 3d), the three‐way valve connecting to the balloon passage was opened, and 1.5 mL air was injected into each balloon using the syringe. The diameter of the balloon after air injection was approximately 1 cm (Figure 3e), and the valve was closed to keep the balloon inflated. The balloon catheter was slowly pulled back, and the two balloons moved towards the gastric cavity. The balloon then drove the metal clips towards the gastric cavity, causing the clips to move gradually towards the gastric cavity (Figure 3f), until the posterior balloon completely entered the gastric cavity. At this time, the two arms of the metal clips and the grabbed gastric wall tissues, together with the nylon rope, were all caught between the anterior and posterior balloons (Figure 3g). The metal clips were placed towards the gastric cavity, and the nylon rope was pre‐retracted to shrink the nylon loop. Air in the posterior balloon was then withdrawn (Figure 3h), whereas the anterior balloon remained inflated, which prevented the metal clips from flipping towards the abdominal cavity (Figure 3i). After reconfirming that the metal clips were not everted or disorganized, the air in the anterior balloon was withdrawn using a syringe, and the balloon was withdrawn (Figure 3j). Finally, the nylon loop was tightened to close the perforation (Figure 3k,l). The operation process is illustrated in Video S1.

### 2.5 Data Collection

The success rate and operation time of the suturing, the number of everted metal clips used, and the number of operations required for successful suturing were collected. Operation time was defined as the time period from the novel balloon‐assisted instrument entering the perforation site to the perforation site being completely closed in the experimental group, and from adjusting the metal clips with the foreign body forceps till the perforation was completely closed in the control group, respectively.

### 2.6 Statistical Analysis

Statistical analyses were performed using the SPSS Statistics version 26.0 (IBM, Armonk, NY, USA). The Kolmogorov–Smirnov test was used to examine whether the continuous variables conformed to a normal distribution. Normally distributed continuous variables were expressed as mean ± standard deviation, whereas non‐normally distributed continuous variables were presented as median and interquartile range (IQR), and between‐group comparisons were conducted using the Wilcoxon signed‐rank test. Categorical data were expressed as numbers and percentages or frequencies, with between‐group comparisons performed using the Fisher's exact test. Statistical significance was set at a two‐tailed *p* value of less than 0.05.

## 3 Results

### 3.1 Description of the Perforation Sites

Twelve perforation sites were made in the two porcine stomachs (10–20 mm in diameter for each) at the upper, middle, and lower parts of the gastric body, the lesser curvature, gastric fundus, and the gastric antrum, respectively.

### 3.2 Outcomes of Perforation Models Generated in the Ex Vivo Porcine Stomachs

The median operation time for physician A was 56.50 s (IQR 40.50 s, 134.50 s) in the experimental group and 215.50 s (IQR 63.75 s, 254.75 s) in the control group, respectively. While those for physician B were 53.00 s (IQR 38.50 s, 87.75 s) and 174.00 s (IQR 104.50 s, 279.25 s) in the experimental and control groups, respectively (Table 1). The differences in operation time between the experimental and control groups were statistically significant for both physicians A (*p* = 0.010) and B (*p* = 0.004).

**TABLE 1:** Suturing operation of artificial perforations in the experimental and control groups.

Items (median [IQR])	Physicians	Control group (*n* = 12)	Experimental group (*n* = 12)	*Z*	*p* value
Suture operation time (s)	Physician A	215.50 (63.75, 254.75)	56.50 (40.50, 134.50)	−2.589	0.010
	Physician B	174.00 (104.50, 279.25)	53.00 (38.50, 87.75)	−2.903	0.004
The number of suture attempts required	Physician A	2 (1, 3)	1 (1, 1)	−2.226	0.026
	Physician B	3 (2, 3)	1 (1, 1)	−2.762	0.006
The number of everted metal clips	Physician A	0 (0, 1)	0 (0, 0)	−2.000	0.046
	Physician B	1 (0, 1)	0 (0, 0)	−1.890	0.059

Abbreviation: IQR, interquartile range.

The median number of suture attempts was 1 (IQR 1, 1) and 2 (IQR 1, 3) for physician A, and 1 (IQR 1, 1) versus 3 (IQR 2, 3) for physician B in the experimental and control groups, respectively. These differences were statistically significant for both physicians (*p* = 0.026 and 0.006, respectively).

The median number of everted clips was 0 (IQR 0, 0) versus 0 (IQR 0, 1) for physician A, and 0 (IQR 0, 0) versus 1 (IQR 0, 1) for physician B in the experimental and control groups, respectively. The difference was statistically significant for physician A (*p* = 0.046), but not for physician B (*p* = 0.059). The overall suture success rate was significantly higher in the experimental group than in the control group (100% [24 out of 24] vs. 75.0% [18 out of 24], *p* = 0.022).

## 4 Discussion

Endoscopic minimally invasive resection has been increasingly used worldwide. The indications of endoscopic minimally invasive resection have gradually expanded from intraluminal conditions to extraluminal GI diseases. Various techniques have been applied in clinical practice, from large medical centers to grassroots hospitals. Endoscopic surgery often causes full‐thickness defects of the GI tract wall either actively or passively. Currently, the most commonly used full‐thickness suture methods include metal clip closure, special instrument suturing, assisted traction technique, and metal clip combined with nylon rope purse‐string suturing. OTSC is the most commonly used special suture instrument. Although OTSC suturing of perforation is secure and easy to operate, the instrument does not easily fall off after the perforation heals, and the cost of the instrument is high; therefore, its clinical application is currently limited to some extent [1, 2, 3, 4, 5, 6, 7]. The single‐clamp channel endoscopic purse‐string suture technique for the treatment of perforation has been used in clinical setting for nearly two decades and is an effective and safe method accepted by a majority of endoscopists [5, 6, 8, 9, 10].

The traditional adjustment method is ineffective when adjusting metal clips to flip towards the abdominal cavity, and often, only one metal clip can be adjusted at a time. In previous studies, technical development focused mainly on improving and updating suture methods, and few studies have addressed the issue of how to quickly and effectively adjust the direction of the metal clips during the suturing process; therefore, we invented this endoscopic purse‐string suture auxiliary instrument trying to solve this issue.

In this study, we utilized ex vivo porcine stomachs to develop the full‐thickness gastric defect model, with varying defect dimensions and anatomical locations. Moreover, a novel endoscopic purse‐string suture auxiliary instrument was applied to facilitate the closure procedure, which achieved a shorter operation time compared with the traditional operation (56.50 s vs. 215.50 s for physician A; 53.00 s vs. 174.00 s for physician B) with statistical significance (*p* = 0.010 and 0.004, respectively). The balloons facilitated clip orientation through frictional force, directing the clips towards the gastric cavity. The circumferential surface of the balloon enables simultaneous adjustment of multiple clips, while the dual‐shell design provides structural support and positional stability throughout the procedure. This prevents unintended clip reorientation during suturing, enabling efficient single‐pass operation. The experimental group achieved a significantly higher suture success rate compared with the control group (100% vs. 75.0%, *p* = 0.022). Notably, failed sutures in the control group were all successfully performed following the implementation of the auxiliary instrument. Also, the number of attempts required for successful closure was significantly lower in the experimental group for both physicians, indicating that the device can effectively improve the efficiency of suturing. We further revealed that the median number of the everted metal clips was 0 and 0 for physician A and 0 and 1 for physician B in the experimental and control groups, respectively, which was statistically significant only for physician A (*p* = 0.046), suggesting that the auxiliary device effectively facilitates proper clip orientation and reduces clip eversion during the procedure. The limitations of the study include inherent differences between the ex vivo model and actual clinical conditions. Though porcine stomachs may simulate human anatomical positioning, the model could not fully replicate the confined gastric perforation or the natural organ fixation by the surrounding tissues and ligaments. The tissue mobility during the procedure increased technical difficulty, although operator stress levels were presumably lower than in clinical scenarios.

In the clinical practice of purse‐string suturing, there are several operative difficulties, one of which involves the adjustment of the direction of metal clips before tightening the nylon rope so that all clips do not flip towards the abdominal cavity or collide with each other. This procedure usually involves the use of hot biopsy forceps and foreign body forceps, etc., to assist in adjusting the direction of the metal clips. Because superb clinical skills are required to control the endoscope, the success rate of a single operation is not high, and the procedure is time‐consuming, especially for perforations at special sites or those in large sizes. The auxiliary instrument demonstrates enhanced utility for novice operators, facilitating the suturing process, though its comparative advantages diminish proportionally with increasing operator experience.

Locations of the perforation were determined based on different anatomical locations to simulate the location of common perforation sites in human. In daily practice, purse‐string suturing for full‐thickness resection at the gastric fundus is the most technically difficult [11, 12, 13]; however, we found that the one‐time suturing rate using this novel auxiliary instrument for perforation at the gastric fundus is significantly higher than that using the traditional suture instrument. After three failed sutures in the control group, the suturing procedure could be completed successfully with the auxiliary instrument. For perforation located at sites where suturing is difficult to perform, the auxiliary instrument can still be used effectively and quickly. It is worth mentioning that, due to the need to extend part of the auxiliary instrument outside the gastric cavity, the field of view is limited. If the space outside the cavity is small, for example, if the wall of the stomach is close to other parenchymatous organs, it is possible to use the anterior balloon only to adjust the metal clips, which can shorten the length of the instrument extending outside the gastric cavity and increase the operative convenience.

There were some limitations to this study. Compared with the more experienced physician, this device seemed to be more user‐friendly for operations by less experienced physicians, which needs to be further evaluated in clinical practice to draw a more solid conclusion.

In conclusion, the utility of this endoscopic purse‐string suture auxiliary instrument has shown good feasibility in ex vivo experiments, which makes purse‐string suturing simpler and faster to perform, effectively increasing the suture success rate. This approach is expected to provide effective assistance in minimally invasive endoscopic surgery, such as EFTR and NOTES.

## Conflicts of Interest

Ping Hong Zhou is an Editorial Board Member for *Journal of Digestive Diseases* and was not involved in the editorial review or the decision to publish this article. All authors declare that there are no competing interests.

## Supporting information


**Video S1.** Using the Disposable Endoscopic Purse‐String Suture Auxiliary Instrument. The novel instrument was sent through the endoscopic forceps channel. The balloon was extended into the abdominal cavity from the middle of the perforation. The three‐way valve connecting to the balloon passage was opened, and 1.5 mL of air was injected into the balloon through a syringe. The wall of the balloon drove the metal clip towards the gastric cavity by friction force, making the clips to gradually move towards the gastric cavity. The root of the metal clip stuck on the waistline between the anterior and posterior balloons and was supported by the walls of these balloons. The air was sucked from the posterior balloon. The anterior balloon was still to prevented the metal clip from flipping towards the abdominal cavity. The air was sucked from the anterior balloon. The balloon was withdrawn. The nylon cord loop was tightened to close the perforation.
